# Vitamin C Protects from Impairment of Memory Induced by E-Cigarette Aerosol Exposure

**DOI:** 10.2174/011570159X341759250119141806

**Published:** 2025-05-06

**Authors:** Karem H. Alzoubi, Omar F. Khabour, Nour Al-Sawalha, Enaam M. Al Momany, Anan Jarab, Razan Haddad, Nasr Alrabadi, Mohammad A.Y. Alqudah, Toka K. Al-zoubi, Thomas Eissenberg

**Affiliations:** 1 Department of Pharmacy Practice and Pharmacotherapeutics, University of Sharjah, Sharjah, UAE;; 2 Department of Clinical Pharmacy, Faculty of Pharmacy, Jordan University of Science and Technology, Irbid, Jordan;; 3 Department of Medical Laboratory Sciences, Faculty of Applied Medical Sciences, Jordan University of Science and Technology, Irbid, Jordan;; 4 Department of Clinical Pharmacy and Pharmacy Practice, Faculty of Pharmaceutical Sciences, The Hashemite University, Zarqa, Jordan;; 5 College of Pharmacy, Al Ain University, Abu Dhabi, United Arab Emirates;; 6 AAU Health and Biomedical Research Center, Al Ain University, Abu Dhabi, United Arab Emirates;; 7 Faculty of Pharmacy, Jordan University of Science and Technology, Irbid, Jordan;; 8 Department of Pharmaceutical Sciences, Faculty of Pharmacy, Jadara University, Irbid, Jordan;; 9 Department of Pharmacology, Faculty of Medicine, Jordan University of Science and Technology, Irbid, 22110, Jordan;; 10 Department of Psychology, Virginia Commonwealth University, Richmond, VA, USA;; 11 Center for the Study of Tobacco Products, Virginia Commonwealth University, Richmond, VA, USA

**Keywords:** Vitamin C, E-cigarette, memory impairment, aerosol, oxidative stress, tobacco

## Abstract

**Introduction:**

E-cigarettes (EC) have been shown to impair memory by disrupting the balance involving ROS and antioxidant enzymes, leading to oxidative stress. Vitamin C (VitC) is a strong antioxidant with cell protective efficacy and scavenges free radicals. The present study evaluated VitC for potential protective effects against EC-induced memory impairment in rat models.

**Methods:**

The animals were exposed to EC for 2 hr/day, with a one-hour break in between, for five days per week over four weeks. Simultaneously, animals were administered Vitamin C at 100 mg/ kilogram/bw/day *via* oral gavage five days/week/for four weeks. After the treatment and exposure period concluded, spatial learning and memory were evaluated using the Radial Arms Water Maze. Furthermore, the oxidative stress biomarkers levels (GSSG, GSH, GSH/GSSG, TBARS, Catalase, and GPx) and brain-derived neurotrophic factor (BDNF) were measured in the hippocampus tissues.

**Results:**

The findings indicated that EC had a detrimental effect on the short-term and long-term memory of the animals (*p* < 0.05). Additionally, EC decreased the levels of GPx, SOD, GSH, the GSH/GSSG ratio, and BDNF (*p* < 0.05). Furthermore, the GSSG level was significantly elevated (*p* < 0.05) by EC. However, Vitamin C prevented impairment of memory and restored levels of biomarkers of oxidative stress and BDNF.

**Conclusion:**

To summarize, exposure to EC resulted in impairments of memory, both short-term and long-term. However, the administration of Vitamin C prevented these negative effects by its antioxidant properties.

## 1 INTRODUCTION

Tobacco use is widely recognized as a significant threat to global health [1, 2]. In 2016, waterpipe and electronic cigarettes (e-cigarettes or ECs) were classified as tobacco products by the FDA, mandating their regulation to be similar to tobacco cigarettes [2]. They were first introduced to the world market in the mid to late 2000s and were marketed as a less harmful or ‘safer’ substitution to conventional smoking of tobacco [1-4]. Globally, data is limited on the number of individuals using ECs [1]. Jerzyński *et al*. utilized established epidemiological calculations to estimate that the global usage of electronic cigarettes (ECs) among adults reached approximately 68 million in 2020 [5]. EC use is an emerging trend among young people due to several reasons, which include the misperception of being a harm reduction approach for smokers, the availability of many pleasant flavors, the absence of regulations following their introduction to the wider market, and its use as a potential smoking cessation tool [2-4, 6].

The main components of ECs include a cartridge, tank, or pod filled with fluid (often contains nicotine, humectants such as vegetable glycerin and propylene glycol, and flavorings), a heating source/atomizer to aerosolize the fluid, and a rechargeable battery [2-4, 7]. The liquid heating process, which replaces tobacco combustion in traditional cigarette smoking, produces an aerosol containing fine particulates [2, 3, 7] that may be hazardous [3, 4, 7, 8]. These include reactive oxygen species (ROS), carbonyl compounds (formaldehyde, acetaldehyde, and acrolein), metals (copper, nickel, or silver from the wires and atomizer), and silicate particles [3, 4, 9-11]. Such toxicants have been associated with respiratory injury, cardiovascular risk, increased oxidative stress, mitochondrial dysfunction, vascular endothelial damage, inflammation, and potential carcinogens with an overlap with tobacco smoking toxicities [1, 3, 4, 12]. However, some toxicants introduced by EC use are not found in tobacco smoke [13]. Despite their widespread use among adolescents and adults, the chronic long-term effects of EC use on human health have not been well characterized [3, 4].

EC use causes oxidative stress, as documented in several *in vitro* and *in vivo* studies. *In vitro,* exposure of human airway epithelial cells and lung fibroblasts to EC aerosol increased inflammatory cytokine secretion [11]. In addition, when vascular endothelial cells of humans are exposed to EC aerosol extract-induced ROS, it causes DNA damage, reduced cell viability, and increased apoptosis [14]. Studies on animal models demonstrated increased oxidative stress and inflammation following exposure to EC aerosol, leading to endothelial dysfunction, and this outcome was also very similar to the effects of tobacco smoke exposure [15-18]. Several human studies also showed elevation in biomarkers of oxidative stress and impairment in endothelial function due to acute short-term EC aerosol exposure [15, 19-21].

There is a scarcity of research on the impact of EC aerosol on the brain. Prasedya and his colleagues conducted a study to evaluate the effect of short-term exposure to EC aerosol on the brain in living organisms. They also compared these effects to those caused by cigarette smoke [22]. They demonstrated that mice exposed to cigarette smoke and EC aerosol exhibited diminished cognitive spatial learning abilities and memory functions. Furthermore, the cerebral cortex histology examinations in mice brains from the group exposed to EC revealed the presence of inflammatory reactions, as indicated by elevated levels of TNF-α as a proinflammatory cytokine and the occurrence of necrosis. Research indicates that aerosol from electronic cigarettes (EC) may potentially impact mood, cognitive abilities, and memory functions in the brain, as well as potentially lead to drug dependence [23]. We have recently shown that EC aerosol exposure negatively affects memory (both short-term and long-term) in rats and leads to changes in brain chemistry, as indicated by oxidative stress biomarkers [24].

Vitamin C, also known as ascorbic acid, is a water-soluble antioxidant. It is crucial for the human body’s various physiological activities [25]. It is primarily found in the brain and is responsible for maintaining homeostasis in the central nervous system (CNS). Its antioxidant properties are crucial for preserving the balance of oxidative processes [25, 26]. VitC has a significant role in neurodevelopment and neurotransmission facilitation [27, 28]. It controls the transmission of dopaminergic, glutamatergic, GABAergic, and cholinergic neurotransmitters and the acetylcholine and catecholamines released from synaptic vesicles [28-30]. Vitamin C eliminates ROS made by glutamate, the primary excitatory brain neurotransmitter, thereby preventing and resolving glutamate-induced neurotoxicity [28]. Previous work showed that VitC deficiency can lead to cognitive impairment by impairing glutamate clearance, leading to neuronal excitotoxicity [31]. However, the potential ability of Vitamin C to preserve cognitive dysfunction and oxidative stress caused by EC exposure to aerosol was not studied. Hence, the present research aimed to investigate the hypothesis that Vitamin C’s antioxidant properties can mitigate the harms of exposure to EC aerosol in rats, specifically by preventing oxidative stress and cognitive impairment.

## 2 METHODS

### 2.1 Animals

Adult Wister male rats aged 10-12 weeks weighing 150-200 g were adapted for one week before the start of the study intervention. The animals were obtained from the Animal House of Jordan University of Science and Technology, Irbid, Jordan. Throughout the study, the animals were maintained in an environment with a constant temperature of 25 ± 1°C and a 12-hour cycle of light and darkness. They were given unrestricted access to both food and water. The animals were randomly assigned over four groups (10 rats/group): (i) Fresh air group, where the animals served as the control group. They were kept in exposure chambers with clean air and water (as the vehicle) that was given to this group by oral gavage. (ii) EC-exposed animals were exposed to EC aerosol for two hours a day with one hour of rest in between, five days a week. In addition, this group was given water *via* oral gavage, similar to the fresh air group. (iii) VitC-treated group in which VitC (100 mg/kg/day, Sigma, St. Louis, MO) was given by oral gavage five days a week. (iv) EC-exposed + VitC-treated group. This group was exposed to EC in group “ii” and VitC in group “iii”. The VitC treatment protocol was based on previous studies showing neuroprotective properties for conditions other than EC exposure [32-34]. All treatments were initiated on the same day and continued for four weeks.

### 2.2 EC Exposure Protocol

A whole-body exposure system was used. The EC-exposed group and the EC-exposed+VitC-treated group were held in the exposure chamber connected to the EC aerosol treatment system. Animals were exposed to EC aerosol as described previously [24, 35, 36]. The EC liquid was formulated using propylene glycol (PG) to vegetable glycerin (VG) (analytical grade) and free-base nicotine sourced from Sigma Aldrich (USA). The proportion of PG to VG was 7:3, and the concentration of liquid nicotine was 18 milligrams per milliliter (mg/ml). The proportions and nicotine concentration mentioned here are representative of EC liquids that are commonly found in the market [37]. The EC aerosol was discharged by a 1.8 Ω single coil EC device (Mini protank2, KangerTech, Shenzhen, China). The EC was operated at a power of 5.76 W using a reverse-puffing machine developed by the Aerosol Research Lab of the American University of Beirut [32, 33, 38], resulting in the production of aerosols. The aerosol produced is directed through the mouthpiece of the EC into the exposure chamber, which has dimensions of 38×25×25 cm (length × width × height). The exposure machine was calibrated to generate a single burst lasting four seconds and containing 116.7 mL of air every 10 seconds during the exposure period. During each hour of exposure, approximately 2 milliliters of EC liquid containing around 36 milligrams of nicotine were consumed. During the process of puffing, a pump is activated to forcefully push fresh air into the air inlet ports of EC. The animals in the control group were only exposed to fresh air.

### 2.3 The Radial Arm Water Maze (RAWM)

As Jibril and colleagues [39-41] described, the four groups of animals were subjected to RAWM cognitive tests to evaluate spatial learning and memory.

### 2.4 Hippocampus Dissection

Following the completion of cognitive testing, the rats were euthanized, and their brains were promptly dissected and placed on filter paper that had been soaked in normal saline solution and crushed ice. The hippocampi were extracted from the brain and stored in 1.5 mL tubes in a liquid nitrogen container. Ultimately, the tubes were stored at a temperature of -30°C until oxidative stress biomarkers were measured. The methodology was extensively elucidated by Alzoubi *et al*., 2021 [42].

### 2.5 Oxidative Stress Biomarkers and BDNF Measurements

To assess the oxidative stress biomarkers, the hippocampal tissue was manually homogenized using a pestle appropriate for the 1.5 mL tube. The homogenization was performed in a lysis buffer [43] and a protease inhibitor cocktail from Sigma-Aldrich Corp, MO, USA. The homogenates were centrifugated at 1400 times the force of gravity for 5 minutes at 4 degrees Celsius to separate and eliminate insoluble substances.

Total protein concentration was quantified using a commercially available BioRad kit in Hercules, CA, USA. To measure reduced glutathione (GSH) levels, the homogenates of the tissue were treated with 5-Sulfosalicylic acid. Subsequently, the mixture was centrifuged at a speed of 1000 times the force of gravity for 10 minutes at a temperature of 4°C. The resulting supernatant was then analyzed using the GSH Assay Kit, following the instructions provided by Sigma-Aldrich Corp. The GSSG analysis followed the same procedures as for reduced glutathione (GSH), except that tissue homogenates were pre-treated with 2-vinylpyridine, as specified in the kit manual. The GPx activity was measured *via* Sigma-Aldrich’s cellular activity assay kit (CGP1) through spectrophotometric analysis. The level of the enzyme superoxide dismutase (SOD) was determined by using the SOD assay kit, following the instructions provided by the manufacturer (Sigma-Aldrich, MO, USA). BDNF activity was quantified using a BDNF assay kit from R&D Systems, USA. The ELx800 from the Bio-tek instrument was employed to assess alterations in absorbance and quantify the specific markers.

### 2.6 Statistical Analysis

To evaluate the variations between different groups, two-way ANOVA was succeeded by the post-test of Bonferroni. The statistical tests utilized version 8.0 of Prism software (Graph Pad, LA Jolla, CA, USA). A *P*-value below 0.05 was deemed significant, and data were given as mean ± SEM.

## 3 RESULTS

### 3.1 EC Exposure and EC+VitC Treatment Effects on Spatial Learning and Cognitive Function

**Fig. (1):**
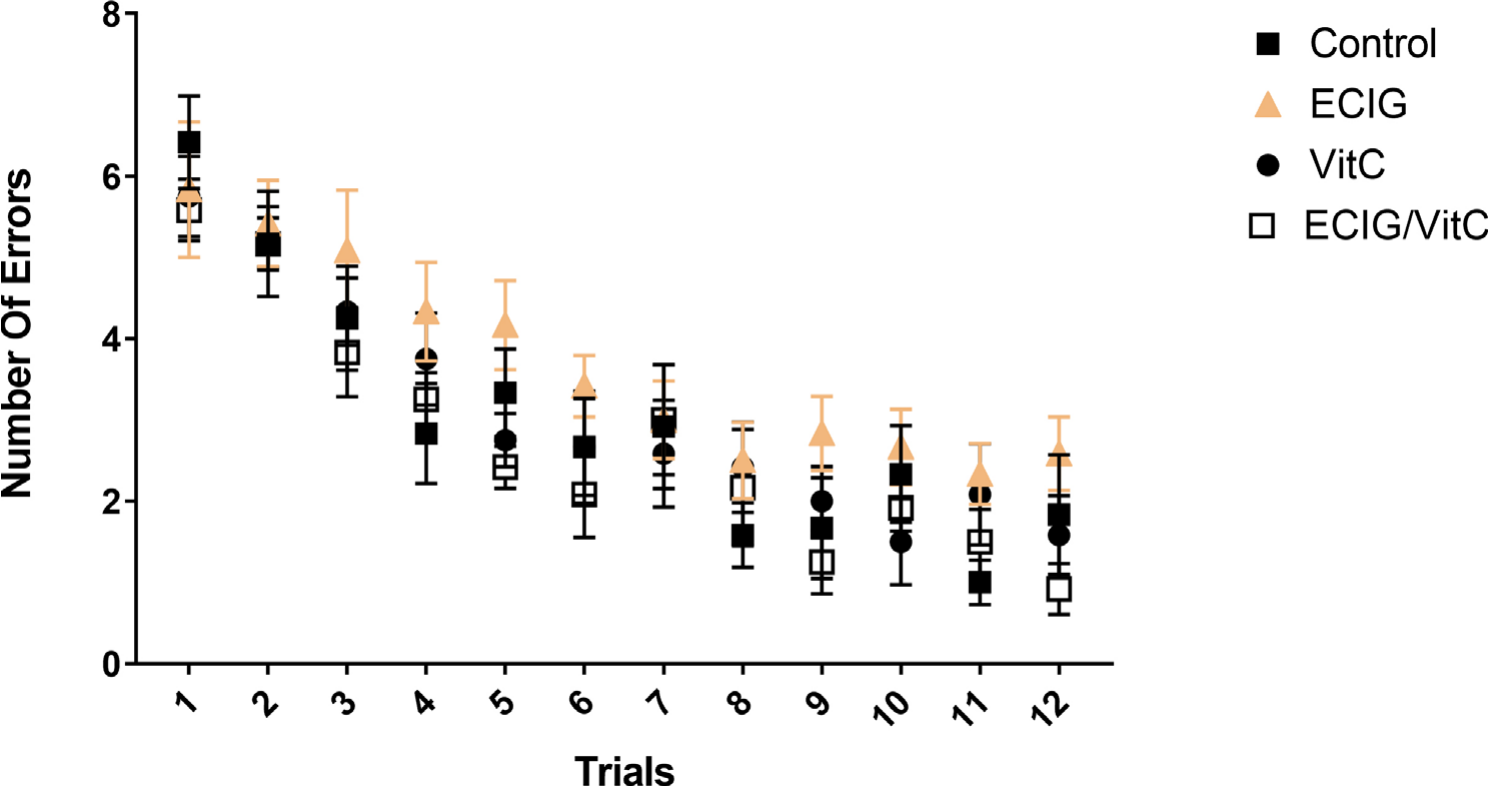
Animals' performance in the acquisition phase. Comparison of e-cigarette exposure (EC), vitamin C (VitC), Control, and EC+VitC. *Indicate *p* < 0.05.

All rats in the four groups committed a high number of errors in the acquisition phase using RAWM. However, the number of errors decreased in the four groups without a significant difference between them as this phase continued for up to 12 consecutive trials (F_(33, 555)_=0.98, *p* > 0.05), and this was evident in the rats’ ability to locate the hidden escape platform (Fig. **1**).

**Fig. (2):**
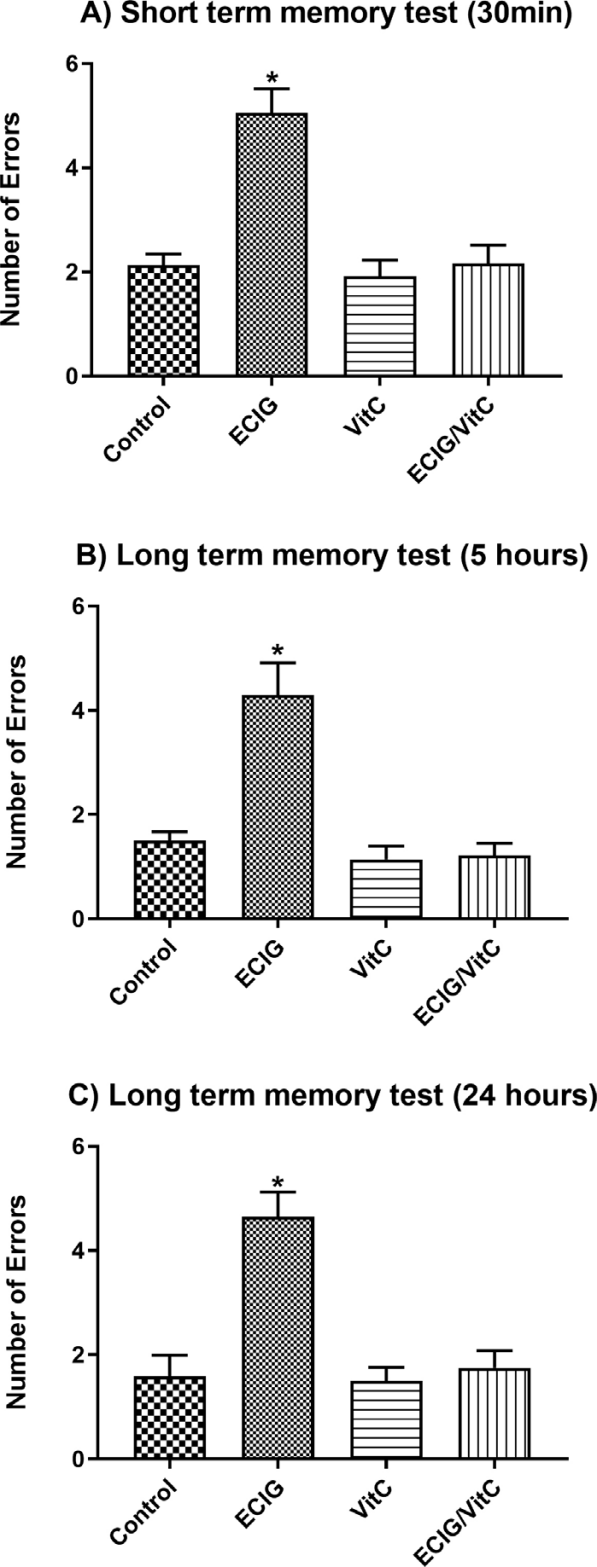
Animals' performance in memory tests. Comparison of e-cigarette exposure (EC), vitamin C (VitC), Control, and EC+VitC. (**A**) Short-term memory test at 30 minutes, (**B**) long-term memory test at 5 hours, and (**C**) long-term memory test at 24 hours. *Indicate *p <* 0.05.

The short-term memory test, which was conducted 30 minutes following the last learning phase trial, showed significant impairment in the EC-exposed group, with greater error numbers than the other three groups (F_(1, 55)_=17.47, *p <* 0.01; Fig. **2A**). On the contrary, the error numbers committed to locating the hidden platform by the EC+VitC-treated group decreased relative to the EC-exposed group. VitC treatment alone (no EC exposure) did not influence results in the short-term memory test, with no significant difference in the error numbers between this group, the EC+VitC group, and the control group (Fig. **2A**).

The assessment of long-term memory was conducted at two time points: 5 hours and 24 hours following the 12th trial of the learning performance test using the RAWM. In comparison to the other three experimental animal groups, rats that were exposed to EC aerosol demonstrated a significantly greater number of errors in the 5- (F_(1, 55)_=15.40, *p <* 0.01, Fig. **2B**) and 24- (F_(1, 55)_=15.49, *p <* 0.01, Fig. **2C**) hour tests following the learning phase. Nevertheless, there was no significant difference in the number of errors observed between the control group and the group exposed to EC+VitC in the 5-hour (Fig. **2B**) and 24-hour (Fig. **2C**) long-term memory tests. There was no significant difference between the VitC-exposed group and the control group at both time points, as shown in Fig. (**2**).

### 3.2 Hippocampal levels of GSH and GSSG and their Ratio

**Fig. (3):**
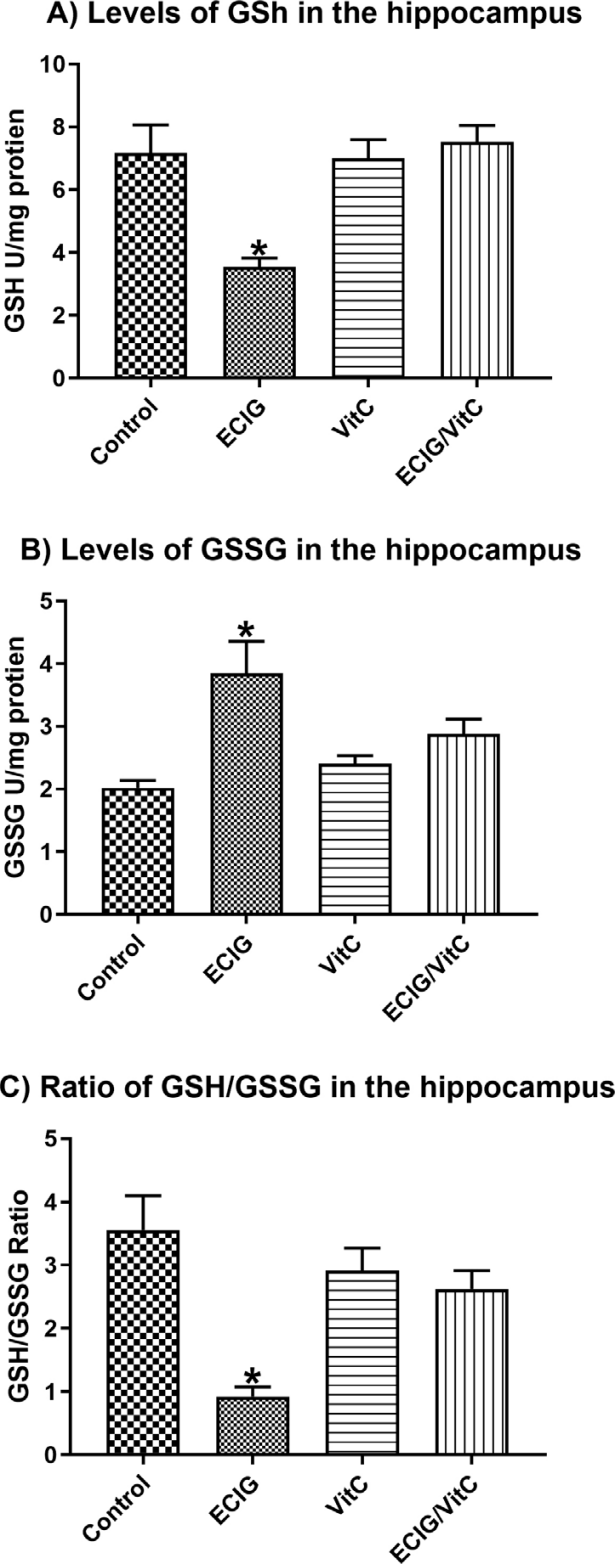
Levels of GSH, GSSG, and ratio of GSH/GSSG. Comparison of e-cigarette exposure (EC), vitamin C (VitC), Control, and EC+VitC. (**A**) Levels of GSH, (**B**) Levels of GSSG, and (**C**) Ratio of GSH/GSSG. *Indicate *p* < 0.05.

Rats exposed to EC aerosol have significantly reduced levels of GSH (F_(3, 58)_=8.28, *p <* 0.01, Fig. **3A**), elevated levels of GSSG (F_(3, 58)_=6.88, *p <* 0.05, Fig. **3B**), and a reduced ratio of GSH/GSSG (F_(3, 58)_=11.40, *p <* 0.05, Fig. **3C**) in comparison to the VitC-treated, EC+VitC-treated, and control groups (*P <* 0.05). The other three groups (*i.e*., control, VitC, and EC+VitC, Figs. (**3A**, **B**, and **C**) had no significant differences.

### 3.3 GPx and SOD Enzymes Activities in Hippocampal Tissue

**Fig. (4):**
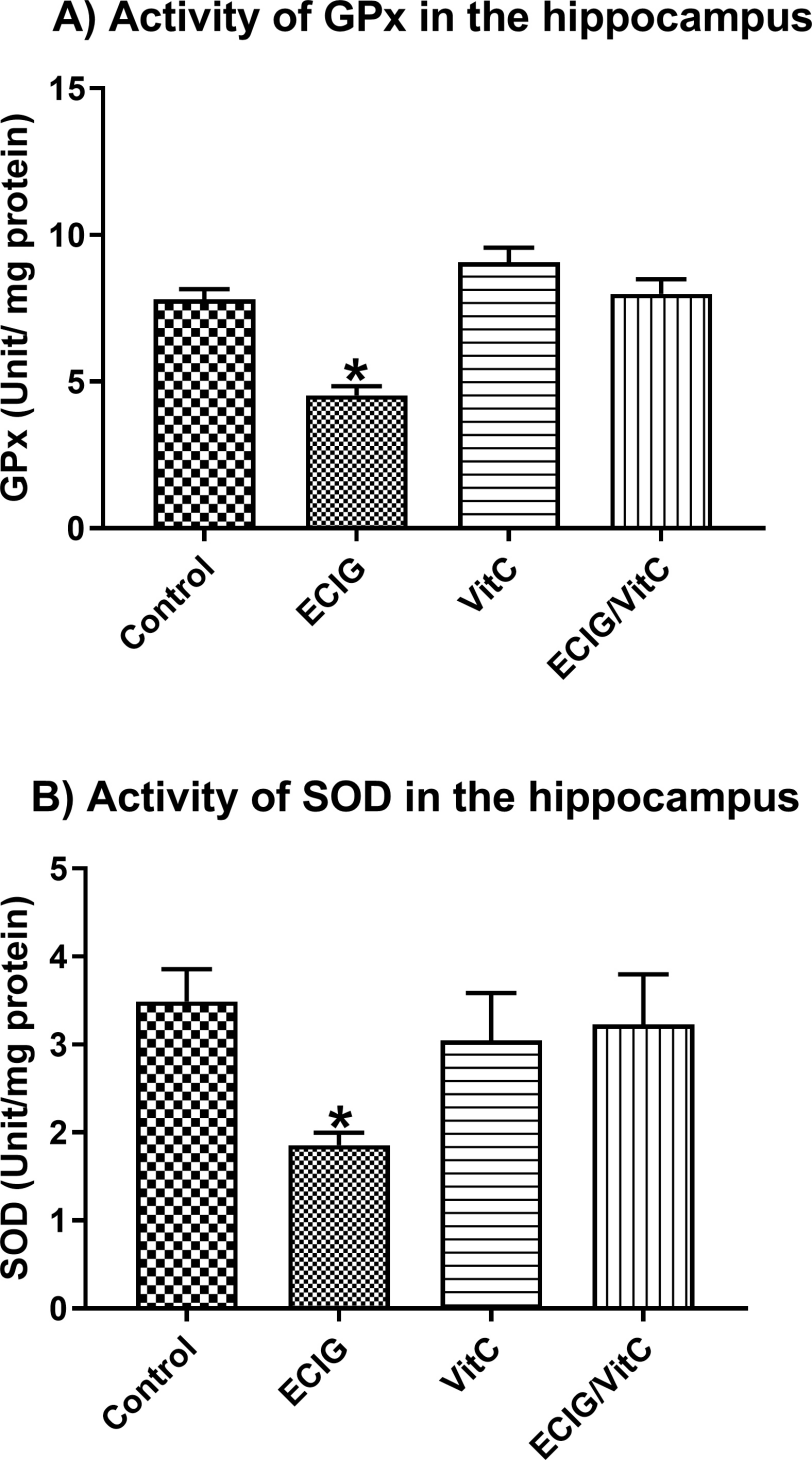
Activities of GPx and SOD. Comparison of e-cigarette exposure (EC), vitamin C (VitC), Control, and EC+VitC. (**A**) Activity of GPx and (**B**) Activity of SOD. *Indicate *p* < 0.05.

The hippocampal antioxidant enzymatic activities of GPx (F_(3, 54)_=10.30, *p <* 0.01, Fig. **4A**) and SOD (F_(3, 58)_=6.60, *p <* 0.05, Fig. **4B**) in the EC-exposed were reduced significantly relative to the other three groups. In contrast, significant differences were not detected among the other three groups.

### 3.4 The Level of BDNF in Hippocampal Tissue

**Fig. (5):**
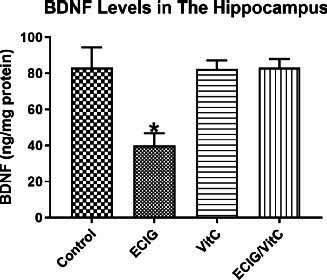
Levels of BDNF. Comparison of e-cigarette exposure (EC), vitamin C (VitC), Control, and EC+VitC. *Indicate *p* < 0.05.

The hippocampal BDNF level in the EC-exposed group was significantly lower compared to the other three groups (F_(3, 58)=_9.97, *p <* 0.01, *p <* 0.05; Fig. **5**). Significant differences were not observed among the other three groups.

## 4 DISCUSSION

Limited research has specifically investigated EC aerosol exposure's effects on oxidative stress and cognitive function. Furthermore, research has not been conducted about the protective impacts of antioxidants, namely VitC, on redox balance and cognitive effects of EC aerosol exposure. Based on this, the current study identifies the impact of VitC on brain oxidative stress biomarkers and cognitive dysfunction induced by EC aerosol exposure in rats. Our findings highlight the potential neuroprotective and therapeutic role of VitC as an antioxidant that mitigates the negative consequences of exposure to EC aerosol on short-term and long-term memory, mainly by reducing oxidative stress in the brain. The present study’s results with simultaneous vitamin C along with EC may not generalize to vitamin C given after exposure. That is, the reactive oxygen species may set in motion neuroinflammation that cannot then be reversed with antioxidants.

### 4.1 EC Exposure and VitC Treatment effects on Cognitive Spatial Learning Ability and Cognitive Memory Function

The brain is highly susceptible to oxidative stress due to the post-mitotic nature of neurons and their high levels of oxygen requirements [44]. Neurodegenerative diseases have been associated with increased compromised antioxidant defenses, resulting in increased ROS levels. These factors result in cognitive decline, oxidative stress in neurons, long-term inflammation, calcium dysregulation, dysfunction of mitochondria, cell damage, hyperactivity of glutamate, and apoptotic effects [25, 28].

Generated ROS can be neutralized by enzymatic (GPx, SOD, catalase, and glutathione reductase) and non-enzymatic antioxidants (VitC, VitE, glutathione, and α-lipoic acid) [28, 45, 46]. VitC scavenges free radicals, reduces oxidative stress, inhibits acetylcholinesterase, which possesses a crucial function in learning and memory, and may help limit the decline of cognitive functions [25, 47, 48]. Therefore, Vitamin C is crucial for restoring or preserving redox homeostasis in oxidative conditions [49]. Vitamin C concentration is elevated in the CNS compared to plasma levels, with the cerebral cortex, hippocampus, and amygdala exhibiting the highest concentrations [27, 50, 51].

This study found that exposure to EC aerosol impaired rats’ cognitive spatial memory in the short- and long-term. At the same time, VitC administration to animals exposed to EC aerosol prevented this negative impact of EC exposure on memory impairments. Previous studies have shown that EC exposure for 1 or 4 weeks has not affected the learning phase of rats [24], and chronic nicotine treatment for 4-6 weeks has normalized impaired learning induced during hypothyroidism in rats [52]. This could be due to nicotine's documented positive effects on the brain's cognitive function [53]. EC exposure for 12 weeks has impaired rats' learning ability [24], which could be explained by nicotinic acetylcholine receptors desensitized to long-term nicotine exposure from EC [54, 55]. In another study [22], a rat’s learning process was negatively impacted by exposure to either ECs or conventional cigarettes for 14 days, which could be explained by the differences in the performed procedures and protocols of the various studies, particularly in terms of the used puffing topography and the time frame of the learning phase (*i.e*., during the 12 trials of training [24] *versus* the next day after training [22]). Our observed VitC effect in protecting animals from ECs-induced memory impairment is consistent with previous findings in PTSD-like behavioral models in rats where VitC administration successfully reduced memory impairment [32]. In addition, we have previously demonstrated that waterpipe smoke exposure causes both short-term and long-term impairment of rats' memory. However, we have also found that administering Vitamin C can prevent this detrimental effect [33]. Multiple other studies have demonstrated markedly reduced blood concentrations of Vitamin C in humans with cognitive decline in comparison to those in good health [27, 56-58]. Furthermore, two cross-sectional studies have shown that a deficiency in Vitamin C may result in neurocognitive dysfunction and is associated with more severe cognitive impairment [59, 60].

### 4.2 Levels and Enzymatic Activities of Hippocampal Oxidative Stress Biomarkers

Consistent with our observations in rats, the inhalation of EC aerosol by mice resulted in an elevation of cytokines that are pro-inflammatory and a decrease in the levels of glutathione in the lungs. These changes are important for keeping the redox stability of the cell [11]. In addition, VitC administration attenuated all changes observed in oxidative stress markers in the PTSD rat model [32]. Moreover, this also agreed with VitC, preventing reduced hippocampal ratio of GSH: GSSG and GPx activity reduced by waterpipe tobacco smoking [33]. Vitamin C’s impact on biomarkers of oxidative stress and cognitive function may be attributed, in part, to the synergistic effects of antioxidants. Other antioxidant regeneration, including glutathione, vitamin E, and catalase, is promoted by Vitamin C [61-63].

### 4.3 The BDNF levels in Hippocampal Tissues

The BDNF is extensively present in the mature mammal brain, with its most abundant concentrations found in the hippocampus [64]. Neurons and synaptic plasticity are regulated by a crucial process involving BDNF that controls their survival, differentiation, growth, and apoptosis [65]. Additionally, it has functions in the cognitive processes of learning and memory in adults [66, 67]. In healthy adults, circulating BDNF may serve as a marker for memory and overall well-being of cognition [68]. Both laboratory studies conducted on cells and animal studies have established a connection between Vitamin C and the stimulation of the expression and production of BDNF [69-72].

## CONCLUSION AND STUDY LIMITATIONS

The current study has some limitations. For example, the animals used in the current study are adults with an age of around 10-12 weeks. Older animals could have higher levels of free iron or copper ions that could elicit the pro-oxidant effects of vitamin C. Also, the dose of Vitamin C used in the current study was to prove the pharmacological concept. It was based on previous studies that showed the protective effect of Vitamin C in multiple conditions other than EC exposure [32-34]. Future studies should construct tests for lower Vitamin C doses, which should be based on the present concept study.

In conclusion, the present results showed that chronic vitamin C administration prevented BDNF reduction induced by EC exposure, which aligns with the available evidence. Therefore, vitamin C’s protective effects on neuronal oxidative stress may be due to its impact on enhancing the expression of BDNF.

## Data Availability

Data will be available upon reasonable request *via* e-mailing the corresponding author.

## LIST OF ABBREVIATIONS

CNS: Central Nervous System
ECs: Electronic Cigarettes
PG: Propylene Glycol
ROS: Reactive Oxygen Species
SOD: Superoxide Dismutase
VG: Vegetable Glycerin
